# Hydrogen peroxide (H_2_O_2_) mediated activation of mTORC2 increases intracellular Na^+^ concentration in the renal medullary thick ascending limb of Henle

**DOI:** 10.1038/s41598-021-86678-1

**Published:** 2021-03-31

**Authors:** Nadezhda N. Zheleznova, Vikash Kumar, Theresa Kurth, Allen W. Cowley

**Affiliations:** Department of Physiology, Medical College of Wisconsin, 8701 Watertown Plank Rd., Milwaukee, WI 53226 USA

**Keywords:** Kidney, Cell signalling, Hypertension, Cell biology, Physiology, Kidney

## Abstract

Hydrogen peroxide (H_2_O_2_) production in the renal outer medulla is an important determinant of renal medullary blood flow and blood pressure (BP) salt-sensitivity in Dahl salt-sensitive (SS) rats. The mechanisms and pathways responsible for these actions are poorly understood. Recently, we have discovered that the mTOR complex 2 (mTORC2) plays a critical role in BP salt-sensitivity of SS rats by regulating Na^+^ homeostasis. PP242, an inhibitor of mTORC1/2 pathways exhibits potent natriuretic actions and completely prevented salt-induced hypertension in SS rats. In the present study, we have found that chronic infusion of H_2_O_2_ into the single remaining kidney of Sprague Dawley (SD) rats (3 days) stimulated the functional marker (pAKT^Ser473^/AKT) of mTORC2 activity measured by Western Blot analysis. No changes in mTORC1 activity in OM were observed as determined by pS6^Ser235/236^/S6. Using fluorescent microscopy and the Na^+^ sensitive dye Sodium Green, we have shown that H_2_O_2_ (100 µM added in the bath) increased intracellular sodium concentration ([Na^+^]_i_) in renal medullary thick ascending limbs (mTALs) isolated from SD rats. These responses were almost completely abolished by pretreatment of mTAL with 10 µM PP242, indicating that mTORC1/2 pathways were involved in the H_2_O_2_ induced increase of [Na^+^]_i_. mTAL cell volume remained unchanged (± 1%) by H_2_O_2_ as determined by 3D reconstruction confocal laser scanning microscopy techniques. Consistent with the microscopy data, Western Blot analysis of proteins obtained from freshly isolated mTAL treated with 100 µM H_2_O_2_ exhibited increased activity/phosphorylation of AKT (pAKT^Ser473^/AKT) that was inhibited by PP242. This was associated with increased protein activity of the apical membrane cotransporter Na^+^-K^+^-2Cl^−^ (NKCC2) and the Na/H exchanger (NHE-3). Na^+^-K^+^-ATPase activity was increased as reflected an increase in the ratio of pNa^+^-K^+^-ATPase^Ser16^ to total Na^+^-K^+^-ATPase. Overall, the results indicate that H_2_O_2_ mediated activation of mTORC2 plays a key role in transducing the observed increases of cytosolic [Na^+^]_i_ despite associated increases of basolateral pump activity.

## Introduction

Reactive oxygen species (ROS) are implicated in the renal dysfunction and hypertension and hydrogen peroxide (H_2_O_2_) is a major component of ROS^1,2^. Chronic infusion of H_2_O_2_ in the renal medulla of normotensive Sprague Dawley (SD) rats resulted in sustained hypertension in rats fed a normal salt diet^3^. Physiological levels of interstitially infused H_2_O_2,_ which failed to produce hypertension in salt-resistant consomic SS-13^BN^ rats, produced an enhancement of salt-sensitivity and hypertension when rats were fed a high salt diet^4^. The interstitial concentrations of H_2_O_2_ achieved in those studies were equivalent to those observed in the renal medulla of Dahl salt-sensitive (SS) rats which we have shown exhibit tissue superoxide (O_2_^•−^) and H_2_O_2_ concentrations nearly twice that of salt-resistant consomic SS-13^BN^ control rats (145 vs. 56 nM)^4–6^. The importance of elevated renal tissue H_2_O_2_ in salt-induced hypertension in SS rats was demonstrated by the reduction of blood pressure (BP) with chronic renal interstitial infusion of catalase (H_2_O_2_ scavenger)^4^. Together, these data indicate that renal levels of H_2_O_2_ play an important role in determining BP salt-sensitivity.

It is recognized that ROS can act as a cell signaling molecule in biological systems^7–9^. The main source of ROS in the kidneys are mitochondria and NOX family ROS-generating nicotinamide adenine dinucleotide phosphate (NADPH) oxidases that can be activated by different stimuli, generating production of O_2_^•−^ and H_2_O_2_^10–12^. NADPH oxidase 4 (NOX4) is the predominant NOX isoform expressed in the kidney and is known to produce mainly H_2_O_2_^13–17^. We have found that knockout of the *Nox4* gene in the SS rat resulted in significant protection from renal oxidative stress, renal injury and hypertension^16^.

Despite the recognition of the importance of H_2_O_2_ as a determinant of BP salt-sensitivity and renal injury, the cell signaling mechanisms whereby H_2_O_2_ exerts these actions has remained to be determined. We have recently found that mTOR (mammalian target of rapamycin) related mechanisms are importantly involved in the regulation of Na^+^ homeostasis and salt-induced hypertension in SS rats^18,19^. Daily administration of mTORC1/2 pathway inhibitor PP242 not only completely prevented but also reversed salt-induced hypertension and kidney injury in SS rats^18^. PP242 administered to SS rats produced a rapid and potent natriuretic response whether administered intravenously or into the renal interstitial space while rapamycin (mTORC1 inhibitor) in amounts that blunted hypertension in SS rats failed to produce a natriuresis^18^. Similar findings were reported in mice^20^ and in pigs which possess a renal structure/function resembling that of the human kidney^21^. In other studies, it has been found that the mTORC2 (but not mTORC1) is importantly involved in phosphorylation of SGK1 which in turn stimulates ENaC activity and ENaC-mediated Na^+^ transport in the aldosterone sensitive distal nephrons (ASDN)^22^. It was also found that inhibition of the mTORC1/2 pathway by PP242 abolished insulin-mediated increase in Na^+^ reabsorption in the ASDN^23^.

Given the preponderance of evidence of the involvement of the mTORC2 pathway in renal tubular Na^+^ transport, the present study sought to determine if the antinatriuretic actions of H_2_O_2_ in the kidney were in part mediated by the mTORC2 pathway. This study focused upon the medullary thick ascending limb (mTAL) since this segment of the nephron reabsorbs ~ 25% of the filtered Na^+^ and has been found to transport and reabsorb excess amounts of NaCl in the SS rats compared to salt-resistant Dahl R rats^24,25^. Studies in our laboratory have found that increased mTAL luminal flow results in increases in intracellular and mitochondrial H_2_O_2_, which is dependent on the presence of NOX4 in contrast to NOX2 which could account solely for increases in O_2_^•−^ production^26^.

The renal-specific NKCC2 (Na^+^-K^+^-2Cl—co-transporter 2) is regulated by changes in state of phosphorylation^27^. Although the specific kinases involved in activation of this co-transporter and the phosphorylation sites involved in this activation are still being explored^28^, several recent studies have shown the involvement of SPAK (Ste20- and SPS1-relared proline and alanine-rich kinase), OSR1 (oxidative stress-responsive kinase) kinases^29^, PKA (protein kinase A)^30^ and AMPK (AMP-activated kinase)^31^.

The goal of this study was to determine if the mTOR pathway was involved in the regulation of intracellular Na^+^ in mTAL and the contribution of H_2_O_2_ towards its activation. The inhibitory effect of PP242 upon H_2_O_2_ mediated activation of mTORC2 and intracellular Na^+^ was studied in mTAL isolated from the outer medulla of SD rats.

## Materials and methods

### Experimental animals

Male SD rats (Harlan Sprague Dawley Inc., Madison, WI) were received having been fed a commercially available pelleted diet (5001, Purina Mills, Gray Summit, MO; 0.4% Na). Upon arrival rats were switched to a purified AIN-76A rodent food containing 0.4% NaCl (Customized food, Dyets, Bethlehem, PA) for 7 days prior to the blood pressure (BP) measurements study with free access to water and used at age 7–8 week old. The animal use and welfare adhere to the National Institutes of Health (NIH) Guide for the Care and Use of Laboratory Animals following protocols reviewed and approved by the Medical College of Wisconsin Institutional Animal Care and Use Committee (AUA00000851).

### Chronic measurement of arterial blood pressure and renal interstitial infusion of H_2_O_2_ in the SD rats

For surgical implantation of the medullary interstitial and femoral arterial catheters, rats were anesthetized using 2.5% isoflurane in O_2_. The study was carried out in compliance with the ARRIVE guidelines (http://www.nc3rs.org.uk/page.asp?id=1357). The fluid filled catheter was inserted into the femoral artery for blood pressure (BP) measurement and the renal medullary interstitial catheter was inserted into the outer medulla (OM) area of the kidney for infusion of H_2_O_2_ as described previously^4^. Both catheters were tunneled subcutaneously, exteriorized through the back of the neck, and attached to a swivel at the top of the cage. This allowed the animal to move freely around its cage while being continuously infused. The catheters were implanted into 2 groups of 9-week old male SD rats that had been unilaterally nephrectomized (right kidney) 7 days before the implantation of catheters. Buprenorphine (0.3 mg/kg) was administered post-operatively to provide analgesia and returned to the home cage for a one week recovery period. Rats were maintained in a temperature-controlled room with a 12-h light/dark cycle in metabolic cages for the entire length of the study. Solutions were infused with a syringe pump (Harvard Apparatus) through a 0.22-μm filter (Cathivex, Millipore Corp). The arterial catheter was filled with saline + 1000 U/mL heparin and connected to a pressure transducer (Cobe), which in turn was connected to a pressure amplifier. Pulsatile arterial pressure and heart rate from the amplifier were sent to a digital computer through an analog-to-digital converter and were sampled throughout the entire 24-h period. Heart rate and mean arterial pressure (MAP) were determined from these data samples^32^. MAP was recorded over six consecutive days. After 3 days of stable baseline MAP measurements, H_2_O_2_ infusion was begun at 347 nmol/kg/min and continued for 3 days to produce an interstitial concentration that was shown to enhance BP salt-sensitivity in SS-13^BN^ rats^4^.

### Western blot (WB) analysis of cortical and medullary tissue isolated from the kidney of SD rats after BP measurements

After 3 days of control MAP measurements (saline infusion), the renal interstitial infusion of H_2_O_2_ was started and continued for 3 days (n = 5 rats). Another group of control rats (n = 4) continued to receive saline throughout the 6-day study. Following completion of the study, rats were anesthetized, and cortex and OM were harvested from the kidneys and snap frozen for WB analysis. Tissues were homogenized using Dounce homogenizer in homogenization buffer (HB) (pH 7.4) containing: 0.25 M sucrose, 0.1 M monobasic KH_2_PO_4_, 0.1 M dibasic K_2_HPO_4_, 0.5 M EDTA, 0.8 mM DTT with addition of protease and phosphatase inhibitors. Protein concentrations were measured by Bio-Rad Protein Assay and samples were prepared for WB analysis.

### mTAL isolation for fluorescence microscopy

Rats were anesthetized with pentobarbital sodium (50 mg/kg ip), and the kidneys were perfused to clear the blood with 10 ml of chilled (4 °C) Hanks’ balanced salt solution (HBSS) containing 20 mM HEPES (HBSS-H, pH 7.4). Renal thin tissue strips from the outer medulla (OM) containing mTAL were dissected at 4 °C using the Leica M3Z stereomicroscope, as previously reported^33,34^ and placed on a glass coverslip coated with the tissue adhesive Poly-L-Lysine in HBSS-H for fluorescence imaging as described previously^35,36^. Several coverslips were prepared from each rat. The dissecting bath was exposed to room air (21% O_2_; 159 mmHg), and, when the mTALs were transferred to the imaging chamber, they were maintained at 37 °C (Warner Instruments) throughout the experiment.

### Fluorescence microscopy for determination mTAL intracellular sodium concentration

The mTAL strips attached to cover slips were incubated with HBSS-H containing 10 nM of the Na^+^-sensitive fluorescence dye Sodium Green (Na Green) and 0.05% Pluronic F-127 for 30 min at room temperature (RT). After 3 washes with HBSS-H to remove an excess of Na Green, cover slip was placed to the heated chamber mounted on the stage of an inverted microscope. The number given for each experimental measurement corresponds to a separate rat in all cases. Na Green fluorescence images were obtained using a Nikon TE-2000U inverted microscope equipped with a 60/1.1 water immersion objective lens and a high-resolution digital camera (Photometrics Cascade 512B, Roper Scientific, Tucson, AZ). Excitation was provided by a 175-W xenon arc lamp (model DG-4, Sutter Instrument, Novato, CA) at alternating wavelengths, and emission was controlled using an optical filter changer (Lambda 10-3, Sutter Instrument)^26^. Na Green was excited at 480/40 nm, and emission signal from 522/30 nm was acquired every 10 s. The signals were normalized by subtraction of the first time point value from each data point.

### Determination of mTAL intracellular Na^+^ concentration

Using the Na^+^ sensitive fluorescent dye, Sodium Green (Na Green), the effects of different concentrations of NaCl upon intracellular Na^+^ concentration ([Na^+^]_i_) were determined in freshly isolated mTAL of SD rats. To obtain basal levels of intracellular Na^+^ in the mTAL, Na Green fluorescence was recorded for 3 min in our standard HBSS bath solution. To get a standard curve for Na^+^, changes of fluorescence were determined in SD mTAL bathed with an HBSS solution, in which 150 mM Na^+^ was replaced by Choline as required to maintain constancy of total osmolality since NaCl concentrations were altered to achieve intracellular [Na^+^] levels of 5, 10, 15 and 20 mM/L. Gramicidin (20 µM), a cation-selective, single-filing pore forming antibiotic agent, was used to permeabilize cell membrane to Na^+^. Ouabain (4 mM) was added to the solutions to inhibit Na^+^-K^+^-ATPase activity to enable steady-state conditions to be established inside the mTAL during addition of NaCl. A calibration curve (y = 0.452 + 10.665; R^2^ = 0.84) was established based on responses of mTAL obtained from four mTAL isolated from SD rats and was used to convert fluorescent units to intracellular Na^+^ concentrations in our study.

### Dose–response curve for H_2_O_2_

H_2_O_2_ dose–response studies were carried out in mTAL isolated from SD rats by Na Green fluorescence recorded for 2 min to obtain a stable baseline signal. 10 µM of H_2_O_2_ was then added to the bath, followed in 1 min by additional concentrations of H_2_O_2_ (30, 50, 70, 100, 500 µM and 1 mM, which on the graph were shown as cumulative concentrations of H_2_O_2_ added to the bath HBSS (10, 40, 90, 160, 260, 760, 1760) to select an appropriate dose H_2_O_2_ for application in the present studies. We found that 100 µM of H_2_O_2_ was required to obtain reproducible and stable response so this was the dose used in the current studies.

### Measurements of mTAL area before and 2 min after 100 µM H_2_O_2_

Bright Field (BF) images of mTAL were used to compare changes in mTAL cellular volume (area). 3D images of the mTAL were obtained using a confocal laser scanning microscope system (Nikon A1-R) to obtain images under control conditions (HBSS, no treatments) and after application of 100 µM H_2_O_2_ for 2 min. A Z-stack of 20–25 consecutive focal planes (total mTAL thickness of ~ 30 μm, 1 step is 1 µm; 0.25 micron/pixel) were collected, which allowed reconstruction of the mTAL volume using the Fiji image processing package (ImageJ 1.47v, National Institute of Health, USA).

### Bulk isolation of mTAL for WB analysis

mTAL isolation was carried out using a modification of described methods^37,38^. Rats were anesthetized with sodium pentobarbital (50 mg/kg), the kidneys cleared of blood by retrograde infusion with 10 ml cold saline solution. An equal volume of a collagenase digestion solution (HBSS-H solution, pH 7.4 with collagenase type 2 (200 units/ml)) was then infused (pump speed 4 ml/min). Kidneys were then immediately removed from the rat, maintained at 4 °C while decapsulated and cut in 1 mm transverse slices. The outer medulla part was cut from each slice and incubated in a collagenase digestion solution at 37 °C for 10 min. After 10 min, the digesting tissue was sheared by pipetted up and down to separate the long mTAL segments from other tubular fragments and cells. The tissue suspension was rinsed with 1% BSA/HBSS-H (4 °C) onto nylon sieves (100 µm and then 70 µm), the tubules flushed off the sieve into a conical 50 ml tube. This procedure was done 5–7 times before centrifugation at 200 g for 5 min at 4 °C^39^. The resulting pellet was washed twice with HBSS-H by centrifugation at 200 g for 5 min at 4 °C and resuspended in 600 µl of HBSS-H (no glucose) (pH 7.4).

### mTAL treatment and samples preparation for WB

Equal amount of isolated mTAL was distributed to 3 tubes and incubated at 37 °C for total 20 min using different treatments. HBSS medium didn’t contain glucose, since glucose oxidase uses glucose to produce H_2_O_2_^40^. It was also shown that another monosaccharide—fructose acutely stimulates NKCC2 activity in mTAL^41^. The mTALs were incubated in HBSS either for 20 min (control, no treatments); for 10 min with HBSS prior to addition of final 100 µM H_2_O_2_ for 10 min; or for 10 min with HBSS containing PP242 (10 µM) prior to addition of 100 µM H_2_O_2_ for 10 min. In the end of incubation time, mTAL were spun at 1000 g for 5 min. Solutions were discarded and 100 µl of HB solution was added to each tube containing mTAL pellet. mTAL were sonicated 5–6 times using 60 sonic dismembrator (from Fisher Scientific; power 3). To avoid excessive heating, sonication was performed in short bursts while the sample is immersed in an ice bath. After homogenization of mTAL, protein concentration was determined by Bio-Rad Protein Assay and samples were prepared for WB analysis^42^.

### Western blot analysis

The protein samples were heated at 100 °C for 10 min in 4 × Laemmli Sample Buffer containing 5% of β-mercaptoethanol, run on 4–15% poly-acrylamide gel (15 µg total protein/lane), transferred to PVDF membrane, blocked in 5% non-fat dry milk dissolved in TBST (1xTBS buffer with 0.1% Tween-20) and probed with primary antibodies (Abs) diluted in 3% BSA/TBST overnight (O/N) at 4 °C. After washing in TBST and incubation with secondary Abs diluted in 5% milk/TBST for 1 h at room temperature, proteins were detected by enhanced chemiluminescence (ECL). Equal amount of protein was loaded in each well as determined by Bio-Rad Protein Assay. All antibodies were selected for their mono-specificity and recognition of a single band of predicted molecular weight. The Image Lab program was used to quantify band intensity.

### Reagents and solutions

Hank's Balanced Salt Solution (HBSS, #14025092, Thermo Fisher), HEPES (#H3375, Sigma), 10 × Tris Buffered Saline (TBS, #1706435, Bio-Rad), Bovine Serum Albumin (BSA, #A9647, Sigma-Aldrich), Tween 20 (#P7949, Sigma), collagenase type 2 (#LS004174, Worthington Biochemical), Poly-L-Lysine (#P4707, Sigma), Sodium Green (#S-6901, Molecular probes), Pluronic F-127 (#P3000MP, Molecular probes), Bio-Rad Protein Assay Dye Reagent Concentrate (#5000006, Bio-Rad), 4-x Laemmli Sample Buffer (#161-0747 from Bio-Rad), PVDF membrane (#1620177, Bio-Rad), Gramicidin A (sc-203061A, Santa Cruz), Clarity Western substrate ECL (#170-5060, Bio-Rad), ouabain (#O3125, Sigma,), PP242 (#S2218, Selleck Chemicals). Sucrose (#S7903, Sigma), monobasic KH_2_PO_4_ (#P0662, Sigma), dibasic K_2_HPO_4_ (#P3786, Sigma), EDTA solution (AM9260G, Invitrogen), DTT (1,4-Dithio-DL-threitol, #43815, Sigma), protease (#P8340, Sigma) and phosphatase (#04906837001, Roche) inhibitors. Cell strainers were from BD Falcon: 70 µm (#352350), 100 µm (#752360).

### Antibodies

Protein kinase B (PKB) (also known as AKT; cs-2920S), pAKT^Ser473^ (cs-4060S), NKCC2 (NKCC21-A from diagnostic lab)^43^, pNKCC2^Ser126^ (was a gift from Dr. Mark Knepper; NHLBI^44^), pNKCC2^Thr96/101^ (was a gift from Dr. Pablo Ortiz), αNa^+^-K^+^-ATPase (sc-21712), pNa^+^-K^+^-ATPase^Ser16^ (cs-4020S)^45^. pNHE-3 (MA1-46464, Invitrogen), NHE-3 (Mab3136, Millipore Sigma), pS6 (cs-2211), S6 (cs-2317).

### Statistical analysis

Values are means ± S.E.M. For WB and fluorescence data, significance was determined using a Student’s t-Test. For blood pressure data, statistical significance was analyzed by two-way analysis of variance (ANOVA) for repeated measures. *P* < 0.05 was considered significant.

## Results

### Hydrogen peroxide (H_2_O_2_) activates renal mTORC2 pathway in vivo in SD rats

**Figure 1:**
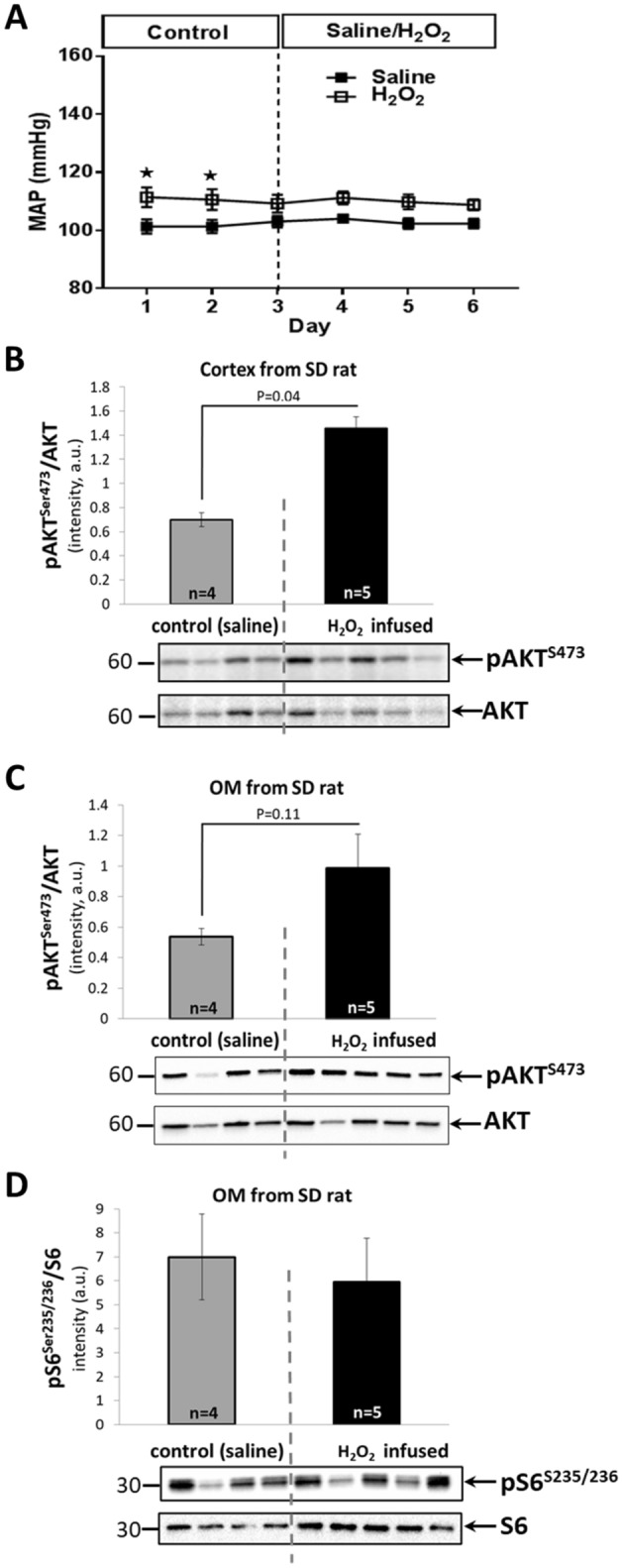
H_2_O_2_ activates mTORC2 pathways in the absence of changes in blood pressure (BP). (**A**) Average 24 h mean arterial pressure (MAP) of single kidney SD rats (n = 9; fed 0.4% NaCl diet) with implanted renal interstitial infusion catheters receiving continuous infusion of isotonic saline (days 1–3) followed by infusion of H_2_O_2_ (347 nmol/kg/min; open squares; n = 5) or continued infusion of saline (closed squares; n = 4). *—small but significant post-surgical differences between control groups on day 1 and 2 (determined by two-way analysis of variance (ANOVA) for repeated measures) were observed which converged to a similar stable BP after several days, at which time the H_2_O_2_ infusion was begun. (**B**, **C**) Western Blot analysis of pAKT^Ser473^ and AKT proteins in renal cortical **(Cortex, B)**, outer medullar **(OM, C)** tissue and pS6^Ser235/236^ and S6 proteins in OM tissue (**D**) homogenates of SD rats fed a 0.4% NaCl diet receiving a renal interstitial infusion of saline (gray bar; n = 4), or after 3 days of H_2_O_2_ (347 nmol/kg/min; black bar; n = 5). * *P* < 0.05 value is shown; determined by Student’s T-test.

To determine the effects of chronic elevation of renal medullary H_2_O_2_ on mTORC2 activity, 3 days of control mean arterial pressure (MAP) measurements were obtained in SD rats followed by continuous intrarenal infusion of either H_2_O_2_ (347 nmol/kg/min) or isotonic saline (control) for another 3 days. Rats were maintained on 0.4% NaCl diet with *ad lib* drinking throughout the study. As shown in Fig. 1A, MAP remained unchanged in both control saline and H_2_O_2_ infused rats. Western Blot (WB) analysis of renal cortical tissue collected at the end of the study showed significant increases in functional marker of the mTORC2 activity (pAKT^Ser473^/AKT)^46,47^ in H_2_O_2_ infused rats compared with saline infused rats (Fig. 1B). WB analysis of renal OM tissue (where mTALs are the most abundant tubules) showed a trend for mTORC2 activation in H_2_O_2_ infused rats compared to saline infused rats, however it did not reach significance (*p* = 0.11) (Fig. 1C). A similar trend was observed in expression of phosphorylated p-Na^+^–K^+^-ATPase (n = 5 rats; *p* = 0.07) but no clear changes were observed in expression of pNKCC2 (n = 5 rats; *p* = 0.14) (data not shown). WB analysis of renal OM tissue didn’t show significant increases (or even tendency) in functional marker of the mTORC1 activity (pS6^Ser235/236^/S6)^48^ in H_2_O_2_ infused rats compared with saline infused rats (Fig. 1D). These data show that non-hypertensive intrarenal elevations of H_2_O_2_ result in activation of the renal mTORC2 pathway in vivo*.*

### Determination of intracellular Na^+^ concentration in mTAL

Using the Na^+^ sensitive fluorescent dye, Sodium Green (Na Green), the effects of changes in intracellular Na^+^ concentration ([Na^+^]_i_) were determined in freshly isolated mTAL of SD rats.

**Figure 2:**
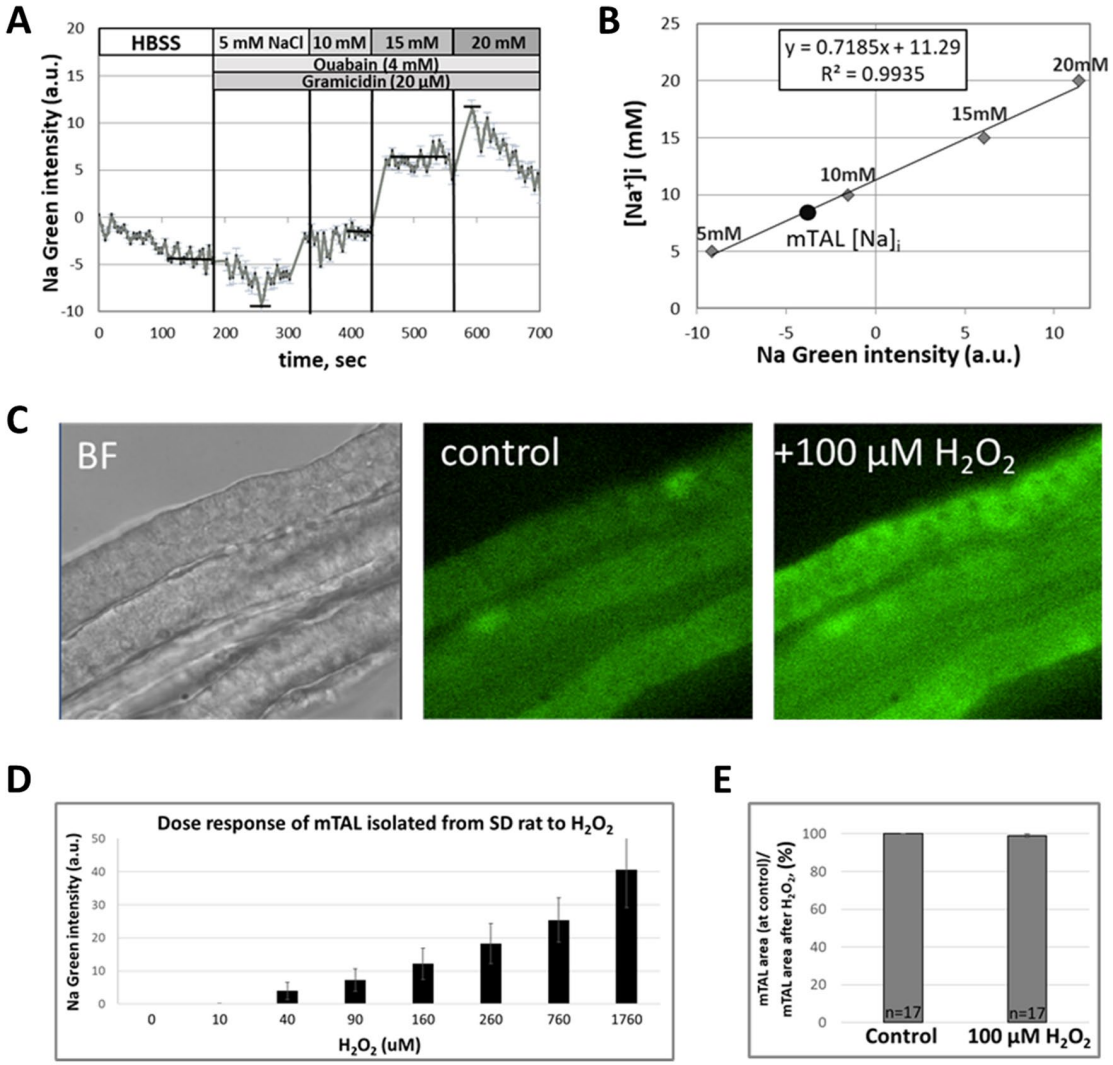
Measurement of intracellular Na^+^ concentrations ([Na^+^]_i_) in mTAL isolated from SD rats. (**A**) Representative tracings of Sodium Green (Na Green) fluorescence normalized to the first point in mTAL permeabilized with Gramicidin (20 µM) followed by addition to the bath of 5, 10, 15 and 20 mM Na^+^ concentrations to create a standard curve. Ouabain (4 mM) was added with Gramicidin to inhibit Na^+^-K^+^-ATPase. (**B**) Representative standard curve of Na Green fluorescence as a function of bath [Na^+^]_i_. (**C**) Representative images of isolated mTAL from SD rat. Left image **(BF)**: bright field image of mTAL. Center image **(control)**: mTAL loaded with Na Green dye for 30 min. Right image **(+ 100 µM H**_**2**_**O**_**2**_**)**: response of Na Green loaded mTAL to application of 100 µM H_2_O_2_ for 5 min. (**D**) H_2_O_2_ dose–response graph (n = 3) measured by Na-Green dye (Na^+^ changes) in mTAL freshly isolated from SD rat. Here is represented the cumulative concentrations of H_2_O_2_. (**E**) Measurements of mTAL area using 3D reconstruction images on confocal microscope before (control) and 2 min after 100 µM H_2_O_2_ application (n = 17 mTAL, 3 SD rats).

A representative trace of Na Green fluorescence and standard curve generated at the end of an experiment are presented in Fig. 2A,B. Intracellular Na^+^ concentration was 8.3 mM in this example as determined using the equation established from the standard curve (y = 0.7185x + 11.29; R^2^ = 0.9935) shown in Fig. 2B. Another calibration curve (y = 0.452x + 10.665; R^2^ = 0.84) was established based on responses of four mTAL obtained from SD rats and was used to convert fluorescent units to intracellular Na^+^ concentrations in our study. This yielded an average [Na^+^]_i_ of 10.8 ± 2.4 mM (n = 4) as determined during the control phase prior to treatment.

### Intracellular Na^+^ responses to H_2_O_2_ application

In each following experiments, baseline [Na^+^]_i_ was recorded for 2 min (control period) with mTAL maintained in the bath HBSS-H solution (140 mM NaCl) prior to addition of H_2_O_2_ to the bath to achieve a concentration of 100 µM, following which [Na^+^]_i_ was recorded over the next 2 min. The representative images of mTAL isolated from SD rat and loaded with Na Green before and after increasing bath H_2_O_2_ concentrations to 100 µM are shown in Fig. 2C.

H_2_O_2_ dose–response studies were carried out in mTAL isolated from SD rat to select an appropriate dose H_2_O_2_ for application in the present studies (Fig. 2D). First, we have applied 10 μM of H_2_O_2_, then added 30 μM (a cumulative dose of 40 μM), then 50 μM (90 μM), 70 μM (160 μM), 100 μM (260 μM), 500 μM (760 μM), 1000 μM (1760 μM). Accumulative concentrations 10, 40, 90, 160, 260, 760 µM and 1760 mM H_2_O_2_ are shown in the Fig. 2D. 100 µM H_2_O_2_ was selected as the stimulus in our study since at this concentration consistent [Na^+^]_i_ responses were observed in nearly all mTAL.

To determine the extent to which observed increases of fluorescence with addition of H_2_O_2_ could be explained by shrinkage of the cell volume we measured mTAL volume in 17 mTALs (3 SD rats) before and 2 min after adding of 100 µM of H_2_O_2_. This analysis found that mTAL volume exhibited less than a 1.0% change with the H_2_O_2_ treatment (Fig. 2E). We have also checked pH after H_2_O_2_ addition to the bath solution (HBSS, pH 7.4) and did not see any pH changes as well.

### PP242 inhibited H_2_O_2_-induced increase of intracellular Na^+^ in mTAL of SD rats

**Figure 3:**
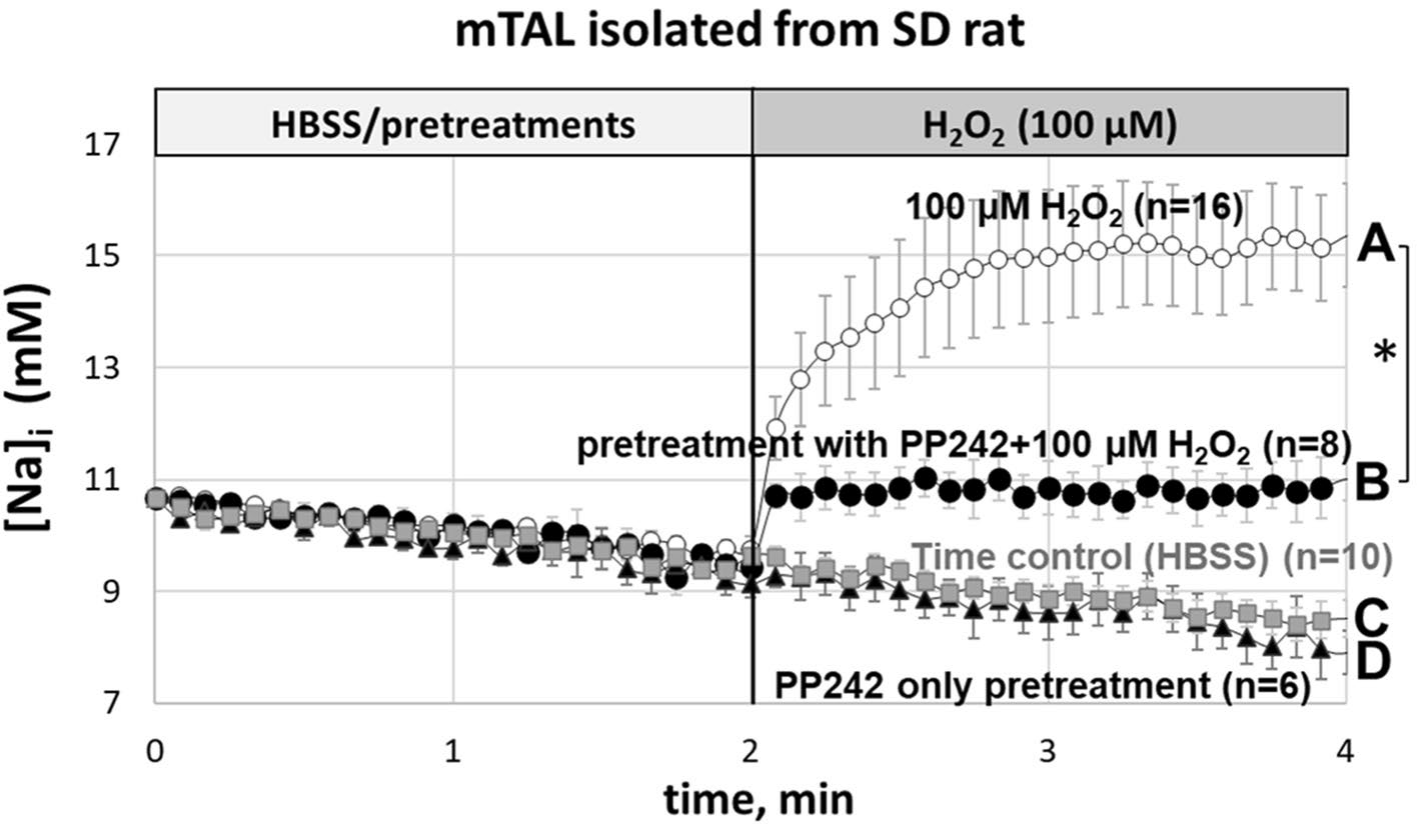
Intracellular [Na^+^]_i_ responses in isolated mTAL determined using Na^+^ sensitive dye, Na Green. (**A**) Responses to 100 µM of H_2_O_2_ (open circles; shown as A on the right; n = 16); (**B**) Responses after pretreatment of mTAL with 10 µM PP242 in HBSS bath for 10 min followed by addition of final 100 µM of H_2_O_2_ concentration (black circles; shown as B on the right; n = 8); (**C**) Time control recording in HBSS medium (gray squares; shown as C on the right; n = 10); (**D**) Pretreatment of mTAL with 10 µM PP242 in HBSS bath for 10 min and throughout all measurements (black triangles; shown as D on the right; n = 6). *P < 0.05 between mTAL treated with H_2_O_2_ compared to those pretreated with PP242; determined by Student’s t-Test.

We have shown using fluorescent microscopy that application of 100 µM of H_2_O_2_ to the isolated mTAL of SD rats significantly increased [Na^+^]_i_ (n = 16 rats, *P* < 0.05) (Fig. 3, curve A). This response was almost completely blocked when mTALs (n = 8 rats) were pretreated with 10 µM PP242 (mTORC1/2 inhibitor) for 10 min (Fig. 3, curve B). Application of PP242 alone (n = 6 rats) for 10 min (Fig. 3, curve D) resulted in a response that did not differ from the HBSS time control responses (n = 10 rats) (Fig. 3, curve C). The small differences observed between the effects of PP242 alone and PP242 plus H_2_O_2_ indicate nearly complete inhibition of the H_2_O_2_ response on mTORC1/2 pathway and can be explained by activation of other signaling pathways or molecules to increase of [Na^+^]_i_ by H_2_O_2._ The effects of vehicle (0.1% ethanol) used to solubilize PP242 were the same as those for HBSS (data not shown).

### Effect of H_2_O_2_ on mTORC2, NKCC2, Na^+^-K^+^-ATPase and NHE-3 activity in mTAL of SD rats

**Figure 4:**
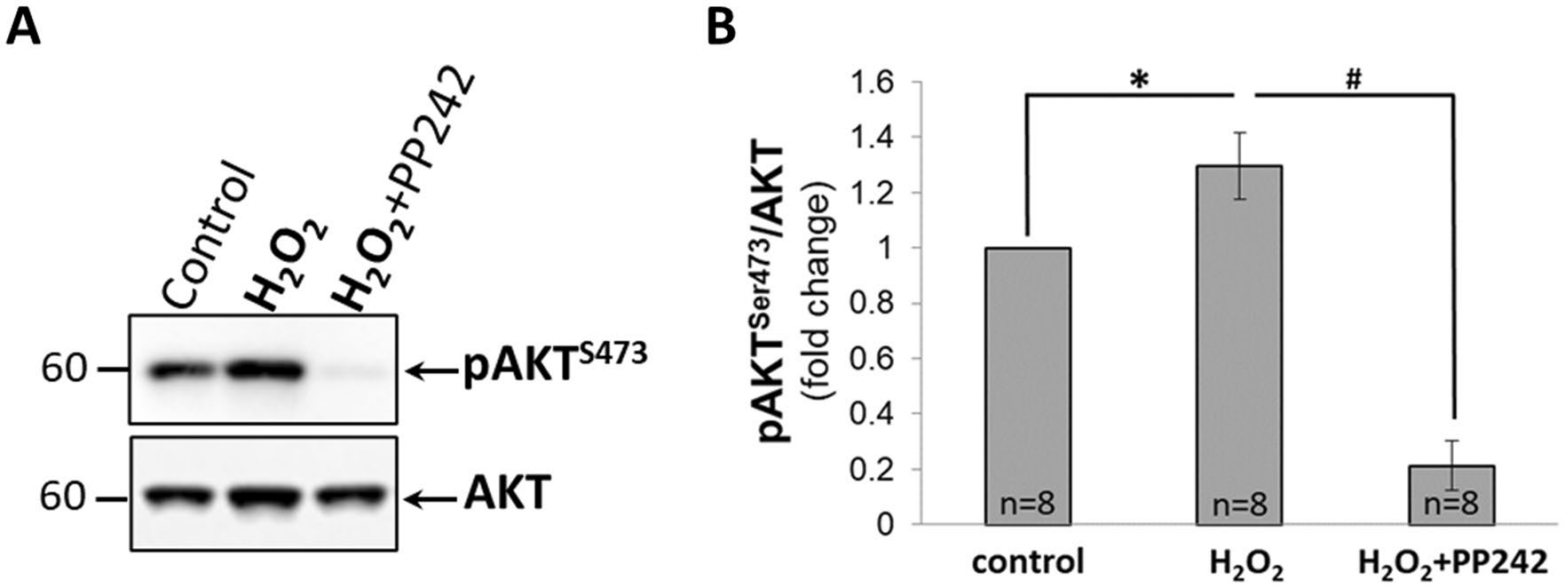
(**A**) Representative Western Blot of total AKT and phosphorylated AKT (pAKT^Ser473^) in mTAL isolated from SD rats. mTAL incubated in HBSS for 20 min (**control**); incubated 10 min in HBSS and then with 100 µM of H_2_O_2_ for another 10 min (**H**_**2**_**O**_**2**_); incubated with 10 µM of PP242 for 10 min and then with 100 µM of H_2_O_2_ for another 10 min (**H**_**2**_**O**_**2**_** + PP242**). (**B**) Activity of mTORC2 determined by densitometric quantification expressed as the ratio of pAKT^Ser473^ relative to total AKT. Data were normalized to control and represented as mean ± S.E.M (n = 8). *P < 0.05 between control and H_2_O_2_ treated mTAL; ^**#**^*P* < 0.05 between H_2_O_2_ treated and H_2_O_2_ + PP242 treated mTAL; determined by Student’s t-Test.

As shown by WB in Fig. 4A, 100 µM H_2_O_2_ increased phosphorylated level of pAKT^Ser473^ protein in mTAL isolated from SD rats. Pretreatment with 10 µM PP242 nearly abolished the amount of pAKT^Ser473^ but had little or no effect on total AKT protein. As summarized in Fig. 4B, 100 µM H_2_O_2_ resulted in a significant increase of mTORC2 activity (ratio of pAKT^Ser473^ to total level of AKT normalized to control; *P* < 0.05; n = 8 rats). This response was virtually abolished by pretreatment with PP242 (*P* < 0.05; n = 8 rats). Together, these data indicate that H_2_O_2_ is acting through the mTORC2 pathway to increase [Na^+^]_i_ in the mTAL of SD rats.

**Figure 5:**
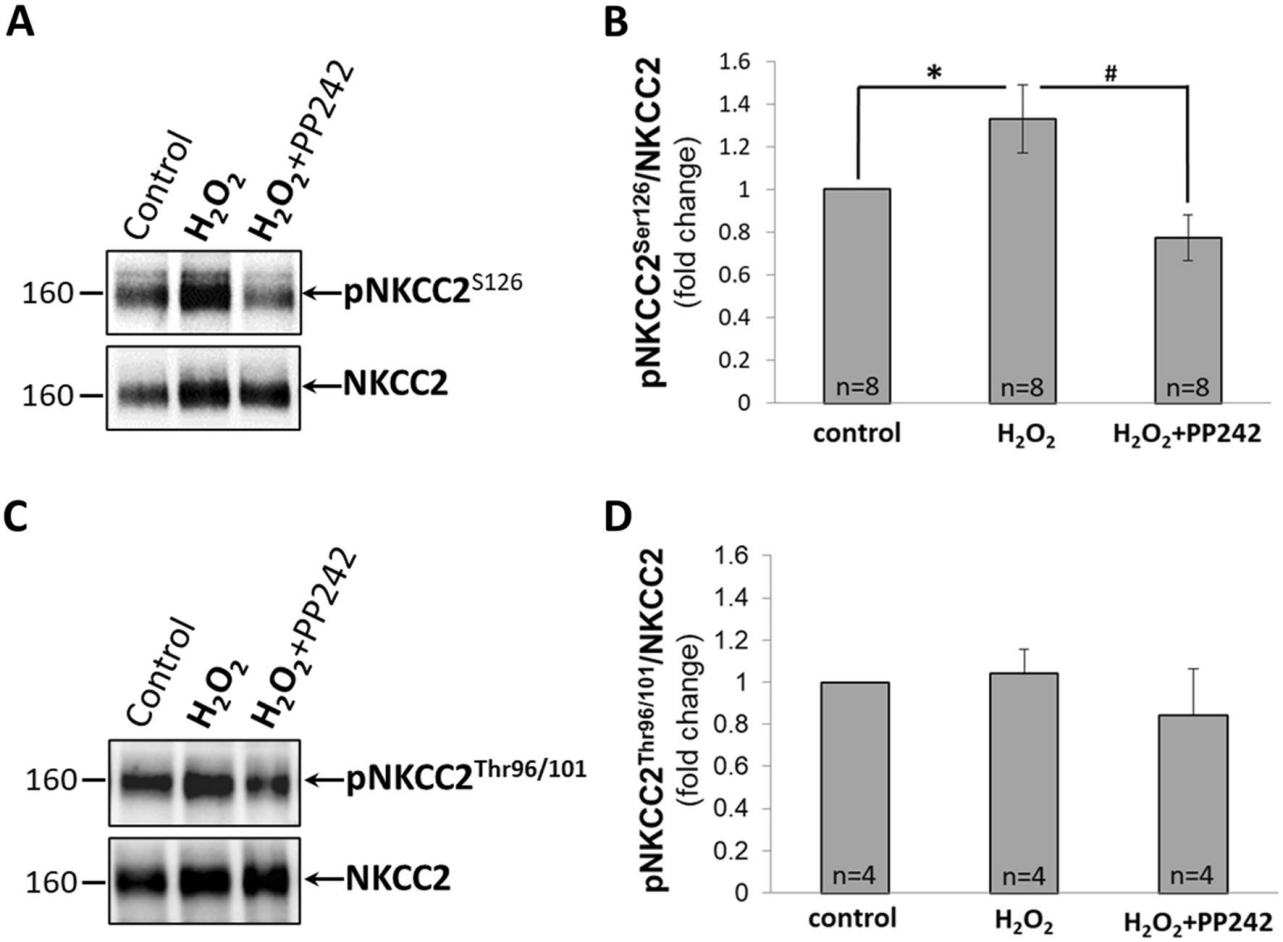
(**A**, **C**) Representative Western Blot of total NKCC2 and phosphorylated NKCC2 cotransporters (pNKCC2^Ser126^ (**A**) and pNKCC2^Thr96/101^ (**C**)) in mTAL isolated from SD rats. mTAL incubated in HBSS for 20 min (**control**); incubated 10 min in HBSS and then with 100 µM of H_2_O_2_ for another 10 min (**H**_**2**_**O**_**2**_); incubated with 10 µM of PP242 for 10 min and with 100 µM of H_2_O_2_ for another 10 min (**H**_**2**_**O**_**2**_** + PP242**). (**B**, **D**) Activity of NKCC2 cotransporters determined by densitometric quantification expressed as the ratio of pNKCC2^Ser126^ (**B**) and pNKCC2^Thr96/101^ (**D**) relative to total NKCC2 (n = 8 and n = 4, respectively). Data were normalized to control and represented as mean ± S.E.M. *P < 0.05 between control and H_2_O_2_ treated mTAL; #*P* < 0.05 between H_2_O_2_ treated and H_2_O_2_ + PP242 treated mTAL; determined by Student’s t-Test.

As shown by WB in Fig. 5A, 100 µM H_2_O_2_ increased protein expression of NKCC2 and pNKCC2^Ser126^ in mTAL isolated from SD rat. Pretreatment with 10 µM PP242 significantly decreased the amount of phosphorylated pNKCC2^Ser126^ but only moderately reduced total NKCC2 protein. As quantified by densitometry, the expression of pNKCC2^Ser126^ relative to total NKCC2 normalized to control (Fig. 5B) was significantly increased by 100 µM H_2_O_2_ (*P* < 0.05; n = 8 rats) and this response was significantly reduced in mTALs pretreated with 10 µM PP242 (*P* < 0.05; n = 8 rats). We have assessed the phosphorylation of NKCC2 at Ser126 since the possibility that H_2_O_2_ can activate NKCC2 channels on this site has not been examined before. Our data indicate that H_2_O_2_ activates NKCC2 cotransporters at Ser126 directly through activation of AKT and not via the SPAK/OSR1 and PKA kinases. Figure 5C,D shows that H_2_O_2_ did not activate NKCC2 by Thr96/101 phosphorylation. We found no significant differences between control non-stimulated and H_2_O_2_ stimulated activation of NKCC2 cotransporters at this site.

**Figure 6:**
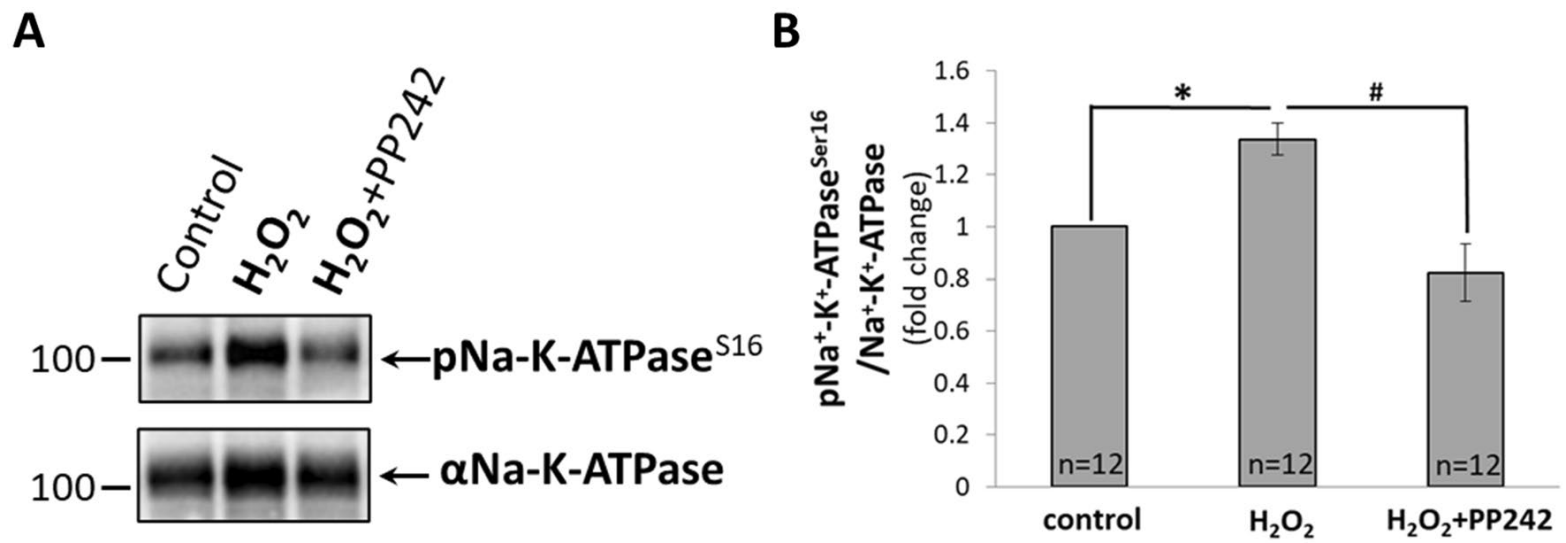
(**A**) Representative Western Blot of total Na^+^-K^+^-ATPase and phosphorylated Na^+^-K^+^-ATPase (pNa^+^-K^+^-ATPase^Ser16^) in mTAL isolated from SD rats. mTAL incubated in HBSS for 20 min (**control**); incubated 10 min in HBSS and then with 100 µM of H_2_O_2_ for another 10 min (**H**_**2**_**O**_**2**_); incubated with 10 µM of PP242 for 10 min and with 100 µM of H_2_O_2_ for another 10 min (**H**_**2**_**O**_**2**_** + PP242**). (**B**) Activity of Na^+^-K^+^-ATPase pump determined by densitometric quantification expressed as the ratio of pNa^+^-K^+^-ATPase^Ser16^ relative to total Na^+^-K^+^-ATPase. Data were normalized to control and represented as mean ± S.E.M (n = 12). *P< 0.05 between control and H_2_O_2_ treated mTAL; #*P* < 0.05 between H_2_O_2_ treated and H_2_O_2_ + PP242 treated mTAL; determined by Student’s t-Test.

WB data show that mTAL treatment with 100 µM H_2_O_2_ was accompanied by significant activation/phosphorylation of Na^+^-K^+^-ATPase (Fig. 6A). As summarized on Fig. 6B, this increase (represented by the ratio of pNa^+^-K^+^-ATPase^Ser16^ to total Na^+^-K^+^-ATPase, normalized to control) was significantly elevated compared to control (*P* < 0.05; n = 12 rats) and was completely inhibited by pretreatment with PP242 (*P* < 0.05; n = 12 rats). These results indicate that H_2_O_2_ activates Na^+^-K^+^-ATPase probably through activation of AKT.

**Figure 7:**
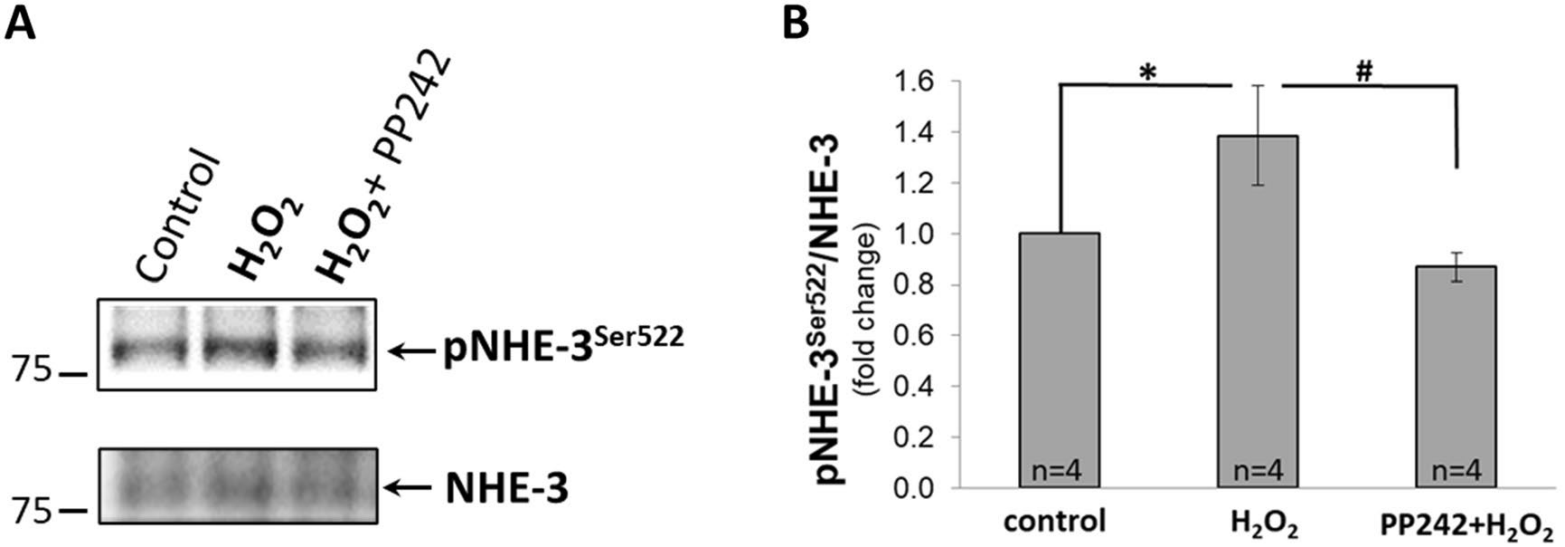
(**A**) Representative Western Blot of phosphorylated NHE-3 (pNHE-3^Ser522^) and total NHE-3 in mTAL isolated from SD rats. mTAL incubated in HBSS for 20 min (**control**); incubated 10 min in HBSS and then with 100 µM of H_2_O_2_ for another 10 min (**H**_**2**_**O**_**2**_); incubated with 10 µM of PP242 for 10 min and with 100 µM of H_2_O_2_ for another 10 min (**H**_**2**_**O**_**2**_** + PP242**). (**B**) Activity of NHE-3 determined by densitometric quantification expressed as the ratio of pNHE-3^Ser522^ relative to total NHE-3. Data were normalized to control and represented as mean ± S.E.M (n = 6). *P < 0.05 between control and H_2_O_2_ treated mTAL, #*P* < 0.05 between H_2_O_2_ treated and H_2_O_2_ + PP242 treated mTAL; determined by Student’s t-Test.

We have shown by WB analysis that mTAL treatment with 100 µM H_2_O_2_ also activates the Na^+^/H^+^ exchanger (NHE-3) on Ser522 (*P* < 0.05; n = 6 rats) (Fig. 7A). As summarized on Fig. 7B, this increase (represented by the ratio of pNHE-3^Ser522^ to total NHE-3, normalized to control) was inhibited by pretreatment with 10 µM of PP242 (*P* < 0.05; n = 4 rats), indicating that this response is via activation of AKT.

Together, these data indicate that elevations of H_2_O_2_ can increase [Na^+^]_i_ in the mTAL of SD rats (Fig. 3) acting through the mTORC2 pathway (Fig. 4) to increase activities of NKCC2 (Fig. 5A,B), Na^+^-K^+^-ATPase (Fig. 6) and NHE-3 (Fig. 7). The relative changes of these transporters cannot be determined in our study to predict Na^+^ flux across the mTAL.

## Discussion

The present study found that chronic 3-day infusion of H_2_O_2_ within the physiological range in the renal medulla resulted in stimulation of the mTORC2 pathway in SD rats. We have shown previously that the elevations of renal medullary H_2_O_2_ obtained with this same dose enhanced blood pressure (BP) salt-sensitivity in salt-resistant rat model (SS-13^BN^ rats)^4^. It was found that application of H_2_O_2_ to freshly isolated mTAL of salt-resistant SD rats resulted in an increase of intracellular Na^+^ and that this response was inhibited by pretreatment with the mTORC1/2 inhibitor PP242. Enhanced phosphorylation of AKT was observed in H_2_O_2_ treated mTAL indicating activation of the mTORC2 pathway. In addition, H_2_O_2_ increased activation of Na^+^-K^+^-2Cl^−^ co-transporter NKCC2 (pNKCC2^Ser126^) and Na^+^/H^+^ exchanger NHE-3 (pNHE-3^Ser522^) in the apical membrane and augmented Na^+^-K^+^-ATPase (pNa-K-ATPase^Ser16^) expression in the basolateral membrane in isolated mTAL. All these events were inhibited by pretreatment with PP242. Together, the results provide novel evidence that H_2_O_2_ can be an upstream regulator of mTORC2 and that stimulation of this pathway could be involved in increased uptake and transport of Na^+^ in the mTAL.

### H_2_O_2_—a novel upstream regulator of mTORC2

The action of H_2_O_2_ on mTOR signaling was the focus of the present study. mTOR is recognized as a key regulator of a wide range of cellular processes including cell growth, survival, metabolism as well as electrolyte homeostasis^49,50^. The biochemistry of the mTOR complexes has been well studied and has shown that mTOR consists of 2 distinct protein complexes, mTORC1 (mTOR complex 1) and mTORC2 (mTOR complex 2) with certain downstream signaling events^49,51–54^. mTORC1 is importantly involved in cell proliferation and immune responses as evidenced in cancer and type 2 diabetes mellitus in humans^20,55–59^.

mTORC2 signaling pathway is recognized to be importantly involved in control of cell proliferation, apoptosis and metabolism^60–62^, but still there are some gaps. Importantly, there is evidence in mice that mTORC2 regulates renal tubular Na^+^ uptake by promoting ENaC activity in distal nephron segments^20^. Others have found actions on potassium transport in mice and concluded that the primary effects of this pathway are upon K^+^ transport in the aldosterone sensitive distal nephrons^57^. Our own studies in SS rats found that inhibition of mTORC2 with PP242 produced a robust natriuresis with no significant effect on kaliuresis when administered acutely or chronically^18^. Remarkably, we found that PP242 not only completely prevented but also reversed salt-induced hypertension and kidney injury in salt-sensitive Dahl S rats^18^.

We have recently reported that H_2_O_2_ at concentration of 100 µM can trigger phosphorylation of AMPK in the normal rat kidney cell line (NRK cells)^63^. Knepper’s group had shown that NKCC2 and NHE-3 were strongly phosphorylated by cAMP^64^. Activation of these proteins by AKT has never been studied. In our present study, using fresh isolated mTAL from SD rat, we have shown that at 100 µM, H_2_O_2_ activates NKCC2 on Ser126 and NHE-3 on Ser522, which is not only due to AMPK activation but also because of AKT activation.

The most novel and physiologically relevant finding of the present study is that elevation of renal medullary interstitial H_2_O_2_ over a period of 3 days activate the functional marker (pAKT^S473^/AKT) of mTORC2. The increased phosphorylation of AKT at Serine 473 indicates that H_2_O_2_ can be an upstream regulator of mTORC2. This was consistent with our findings that H_2_O_2_ stimulated phosphorylation of AKT in freshly isolated mTAL as was observed by others in cultured cells^65,66^.

### H_2_O_2_ enhances phosphorylation of Na^+^ transporters in mTAL

H_2_O_2_ is toxic to cells at high concentrations, which is the key element of the rapid respiratory burst of macrophages to kill bacteria. However, H_2_O_2_ at lower concentrations can serve as an important physiological signaling molecule in both the vasculature^67,68^ and in the kidney^1,3,69^. The role of H_2_O_2_ in modulation of epithelial Na^+^ channel (ENaC) in the collecting duct (CD) through the activity of other signaling molecules has been reported^70–72^. The stimulatory effects of EGF, insulin, and IGF-1 upon ENaC activity in the principal cells of the aldosterone sensitive distal nephron appear to be mediated though local H_2_O_2_ release^73^. Exogenous addition of H_2_O_2_ to the incubation medium of A6 (derived from amphibian distal nephron) cell monolayers caused an increase in PI3-kinase activity (which can activate the mTOR pathway) and a subsequent rise in sodium transport^74^.

SS rats fed a high salt (HS) diet exhibit enhanced Na^+^ reabsorption in the mTAL and this is associated with increased ROS production^1,75^. Studies by our lab have highlighted the important role of renal H_2_O_2_ in salt-sensitive hypertension^4,5,16,36^. Ortiz and Garvin found that HS intake enhanced NaCl reabsorption through increased numbers of surface NKCC2 cotransporters in mTAL isolated from SS rat^76,77^. It is also known that NHE3 transporters account for a significant portion of renal and intestinal Na^+^ absorption^78^. The NHE3 inhibitor S3226 improved renal function after experimental ischemia/reperfusion^79^. Na^+^-K^+^-ATPase activity in mTAL of Spontaneously Hypertensive (SHR) rats was also found to be increased compared to Wistar–Kyoto (WKY) controls in 12 week old rats^80^.

It appears the greatest source of H_2_O_2_ in the renal medulla is NOX4 which produces largely H_2_O_2_^26^. Nisimoto et al. found that approximately 90% of the electron flux through isolated NOX4 produces H_2_O_2_ and only 10% forms superoxide^81^. Saez et al. demonstrated that NOX1/4 inhibitor blunted NKCC2 activity in mTAL of SD rat^77^. Although NOX4 predominantly produces H_2_O_2_, while NOX1 and NOX2 produce predominantly superoxide, NKCC2 cotransporters could be activated by both ROS’s. NOX4 is the most abundant NADPH oxidase in the kidney^82–84^ and we have found that NOX4 and NOX2 mRNA are detected at similar levels the mTAL while NOX1 is expressed at very low levels^14,82^. It is possible that H_2_O_2_ may stimulate production of superoxide from these different sources that could contribute in part to the increase of intracellular [Na^+^] in the present studies. The results of the present study does not attempt to sort the effects of the many variations of free radicals, but specifically shows that H_2_O_2_ acts as a stimulus for increasing [Na^+^]_i_ (Fig. 3) and activation of Na^+^ transporters in mTAL (Figs. 5, 6, 7).

### Physiological relevance and unifying hypothesis of present study

Although it would seem that high concentrations of H_2_O_2_ were needed to elicit Na^+^ responses in the mTAL. This is clearly the case, but it must be recognized that 100 µM concentrations of extracellular bath H_2_O_2_ cannot be extrapolated to the intracellular H_2_O_2_ concentrations in the mTAL. It was found by He-Ping Ma^71^, who measured intracellular H_2_O_2_ in A6 cells derived from normal kidneys of an adult male toad (Xenopus laevis) with the hydrogen peroxide assay kit, that high concentrations of exogenous H_2_O_2_ (~ 3 mM) were required to elevate intracellular H_2_O_2_ to a concentration of ~ 12 nM. This was due to the presence of abundant catalase that rapidly hydrolyzed H_2_O_2_ that entered the cells^71^. We have found high levels of catalase activity in OM tissue of SD rats as was determined by the assay kit obtained from Cayman chemical (#706002). With these high concentrations of catalase, 100 µM of H_2_O_2_ added extracellular will be quickly hydrolyzed inside the mTAL to nM concentrations. This represents nearly 250,000 fold difference between bath and intracellular concentrations illustrating the difficulty of extrapolating bath concentrations to intracellular concentrations. It is also relevant to point out that analysis of human urine by others indicates that H_2_O_2_ urine concentrations are in the range of 100 µM^85,86^ consistent with concentration of H_2_O_2_ added to mTAL in our study.

The results of the present study mechanistically link the anti-natriuretic effects of H_2_O_2_ with the activation of mTORC2 and enhanced Na^+^ retention in the mTAL of SD rats. PP242 treatment produces a potent natriuretic response in SS rats^18^ as found in mice as well^20^. We have shown that increased delivery of Na^+^ to the mTAL enhances H_2_O_2_ production via membrane NOXs and mitochondria^26,33^. The results of the present study demonstrate that elevations of H_2_O_2_ stimulated phosphorylation of AKT (mTORC2 signaling pathway) resulting in an increase of cytosolic [Na^+^]_i_ in the mTAL. This was associated with an increased protein expression of the apical membrane cotransporter Na^+^-K^+^-2Cl^−^ (NKCC2) and the Na/H exchanger (NHE-3) and this in turn with an increase of Na^+^-K^+^-ATPase activity indicated by increase in the ratio pNa^+^-K^+^-ATPase^Ser16^ to total Na^+^-K^+^-ATPase. Although this would serve to offset the rise of cytosolic [Na^+^]_i_ as described by others^87–89^, this response was insufficient to completely buffer the rise of [Na^+^]_i_. Overall, the results indicate that H_2_O_2_ mediated activation of mTORC2 plays a key role in transducing the observed increases of cytosolic [Na^+^]_i_ in mTAL.

## Supplementary Information

Supplementary Figures
