# Supplementary material for: Hydrogen peroxide (H2O2) mediated activation of mTORC2 increases intracellular Na+ concentration in the renal medullary thick ascending limb of Henle
Source: Sci Rep. 2021 Mar 31;11:7300. doi: 10.1038/s41598-021-86678-1 (PMC8012714; doi:10.1038/s41598-021-86678-1)

Hydrogen peroxide (H_2O_2) mediated activation of mTORC2 increases intracellular Na^+ concentration in the renal medullary thick ascending limb of Henle.

Nadezhda N. Zheleznova, Vikash Kumar, Theresa Kurth,

Allen W. Cowley, Jr

Department of Physiology, Medical College of Wisconsin, Milwaukee, WI 53226

Running head: H_2O_2 activates sodium transport in mTAL

Corresponding authors:
Allen W. Cowley, Jr, PhD
Department of Physiology
Medical College of Wisconsin
8701 Watertown Plank Rd.
Milwaukee, WI, 53226, USA
e-mail: cowley@mcw.edu
tel: (414) 955-8277
fax:(414) 955-6546

Nadezhda N. Zheleznova, PhD
e-mail: nzhelezn@mcw.edu
tel: (414) 955-4360

Whole gels

Figure 1B.

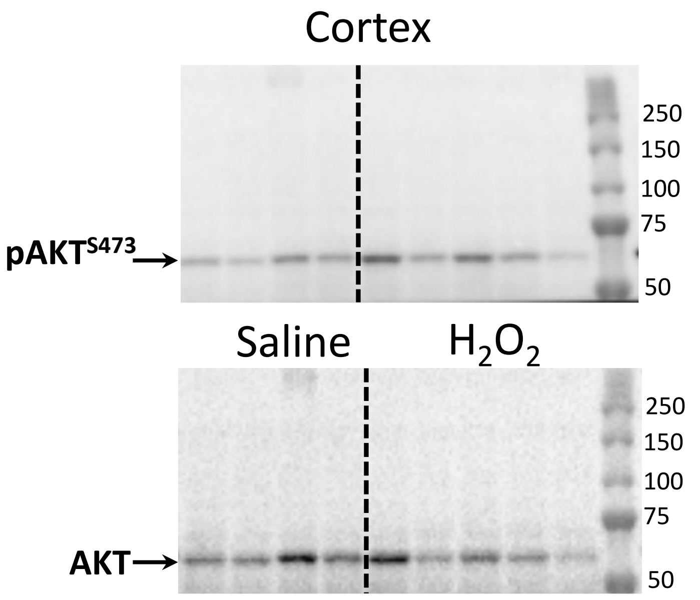

Figure 1C.

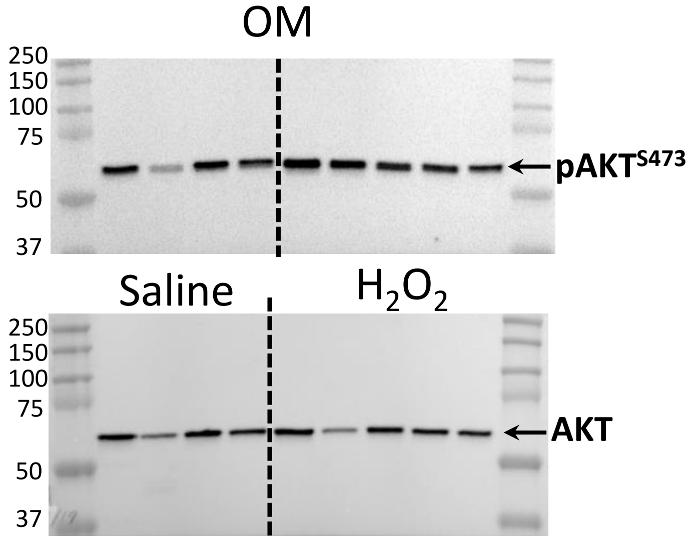

Figure 1D.

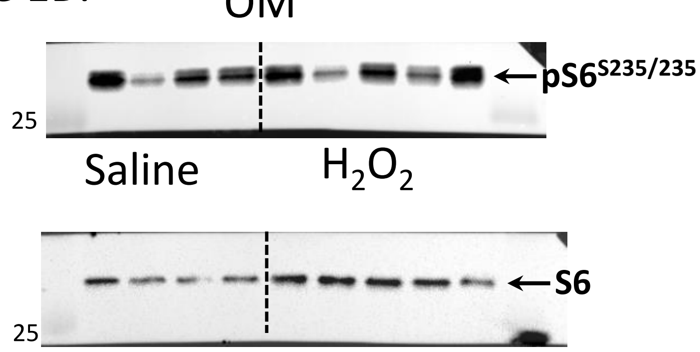

Figure 4A. Shown in the red rectangle - WB from manuscript, other 2 lines are from unrelated to the manuscript different treatments.

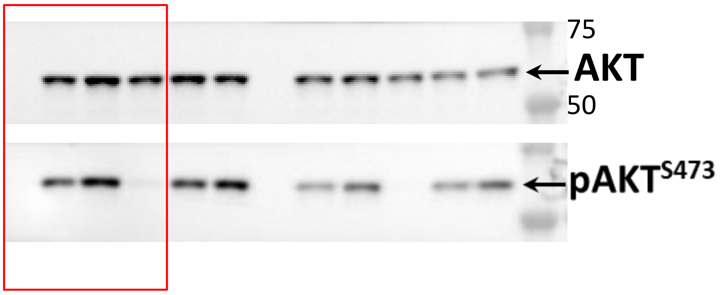

Figure 5A. Shown in the red rectangle - WB for manuscript, other lines are from unrelated to the manuscript different treatments.

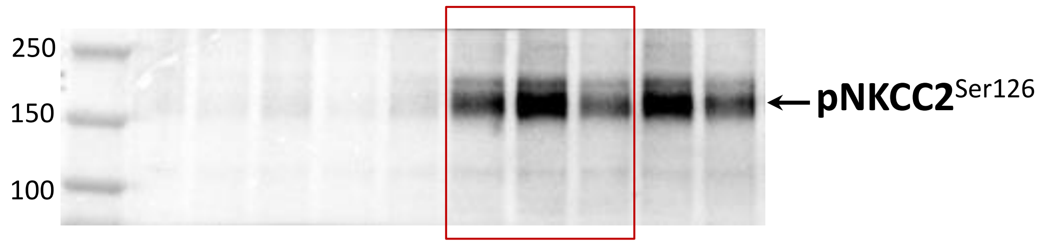

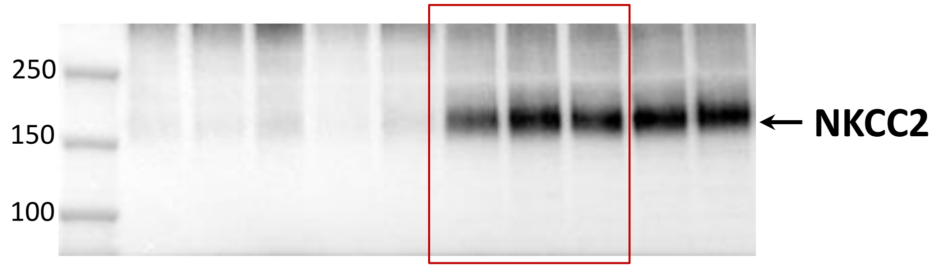

Figure 5C. Shown in the red rectangle - WB for manuscript, other lines are from unrelated to the manuscript different treatments.

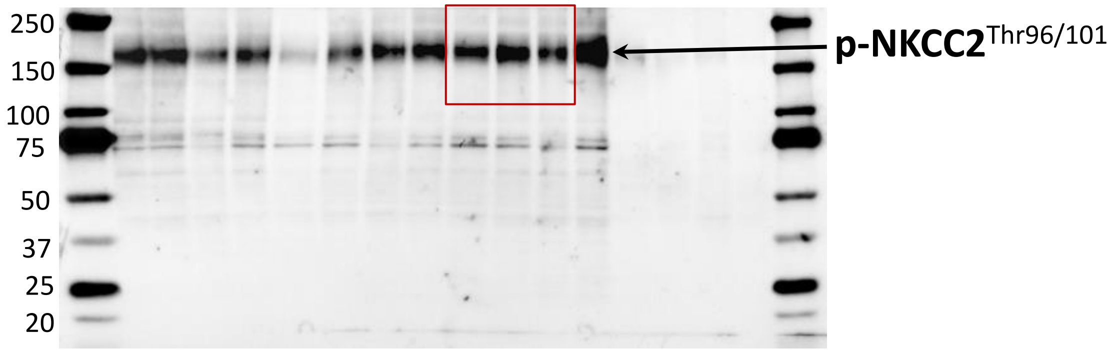

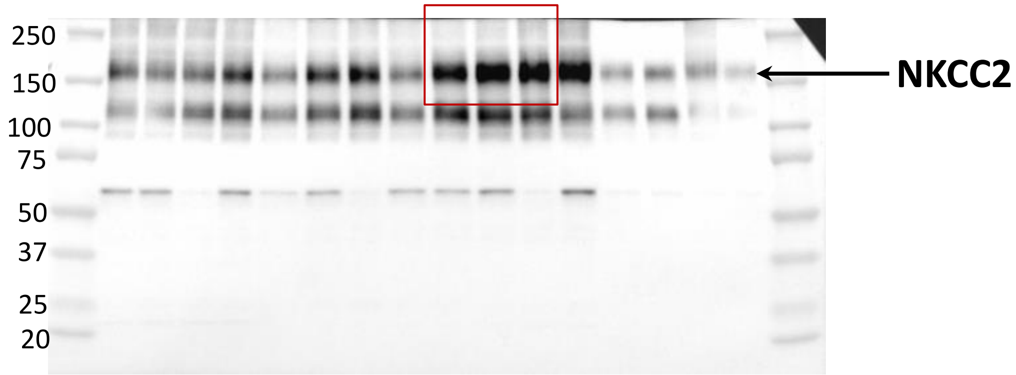

Figure 6A. Shown in the red rectangle - WB for manuscript, other 2 lines are from unrelated to the manuscript different treatments.

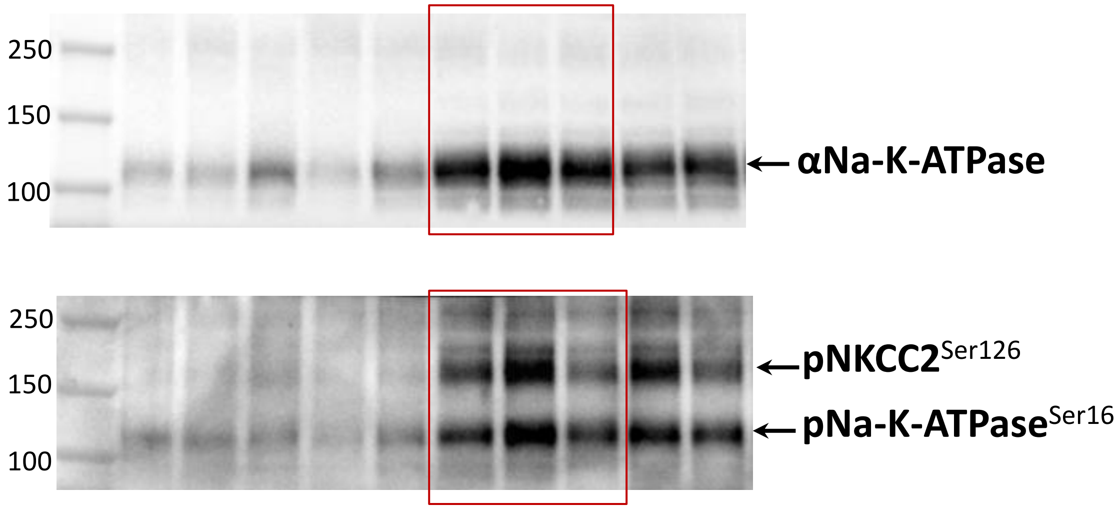

The same WB was stained first with pNKCC2 Abs and then with pNa-K-ATPase Abs

Figure 7A. Shown in the red rectangle - WB for manuscript, other 3 lines are from unrelated to the manuscript different experiments.

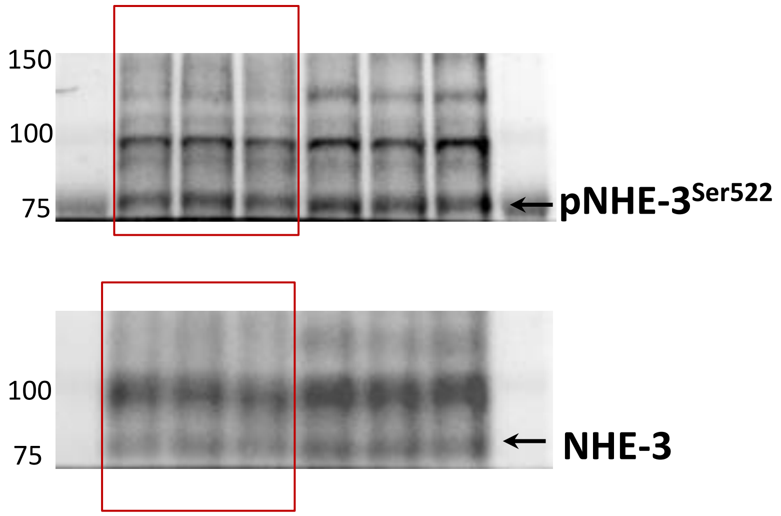

Supplement: Supplementary file 1 — Supplementary Figures [file 41598_2021_86678_MOESM1_ESM.pdf]
